# Biochemical Regulation of the Glyoxalase System in Response to Insulin Signaling

**DOI:** 10.3390/antiox10020326

**Published:** 2021-02-22

**Authors:** Der-Yen Lee, Yu-Chin Lin, Geen-Dong Chang

**Affiliations:** 1 Graduate Institute of Integrated Medicine, China Medical University, No. 91, Hsueh-Shih Road, Taichung 40402, Taiwan; 2 Ph.D. Program for Health Science and Industry, China Medical University, No. 91, Hsueh-Shih Road, Taichung 40402, Taiwan; hsiphd@mail.cmu.edu.tw; 3 Graduate Institute of Biochemical Sciences, National Taiwan University, No.1, Section 4, Roosevelt Road, Taipei 106, Taiwan

**Keywords:** glyoxalase, methylglyoxal, glycation, insulin, metformin, metabolism

## Abstract

Methylglyoxal (MG) is a reactive glycation metabolite and potentially induces dicarbonyl stress. The production of MG in cells is increased along with an increase in carbohydrate metabolism. The efficiency of the glyoxalase system, consisting of glyoxalase 1 (GlxI) and glyoxalase 2 (GlxII), is crucial for turning the accumulated MG into nontoxic metabolites. Converting MG-glutathione hemithioacetal to S-d-lactoylglutathione by GlxI is the rate-determining step of the enzyme system. In this study, we found lactic acid accumulated during insulin stimulation in cells, however, cellular MG and S-d-lactoylglutathione also increased due to the massive flux of glycolytic intermediates. The insulin-induced accumulation of MG and S-d-lactoylglutathione were efficiently removed by the treatment of metformin, possibly via affecting the glyoxalase system. With the application of isotopic ^13^C_3_-MG, the flux of MG from extracellular and intracellular origins was dissected. While insulin induced an influx of extracellular MG, metformin inhibited the trafficking of MG across the plasma membrane. Therefore, metformin could maintain the extracellular MG by means of reducing the secretion of MG rather than facilitating the scavenging. In addition, metformin may affect the glyoxalase system by controlling the cellular redox state through replenishing reduced glutathione. Overall, alternative biochemical regulation of the glyoxalase system mediated by insulin signaling or molecules like biguanides may control cellular MG homeostasis.

## 1. Introduction

Methylglyoxal (MG), an intermediate shunted from glycolysis, is prone to form advanced glycation end products (AGEs) with the amino groups of proteins and nucleic acids, causing dicarbonyl stress. Eliminating an orthophosphate from glycolytic glyceraldehyde-3-phosphate or dihydroxyacetone phosphate is the major source of cellular MG [1]. About 0.1–2% of arginine residues of cellular proteins and 10 ppm nucleobases of cellular DNA could be modified by MG [2]. The glyoxalase enzyme system, consisting of glyoxalase 1 (EC 4.4.1.5; GlxI) and glyoxalase 2 (EC: 3.1.2.6; GlxII), efficiently controls the level of MG. GlxI, a dimeric Zn (II) metalloenzyme, is able to turn the hemithioacetal formed by MG and glutathione into a stable isomeric S-d-lactoylglutathione. Subsequently, S-d-lactoylglutathione is converted to d-lactate and glutathione by GlxII [3,4].

Excessive accumulation of MG may induce modifications of mitochondrial proteins leading to dysfunction of the mitochondria [5] and thus the production of reactive oxygen species (ROS) [6,7,8,9]. Furthermore, GlxI overexpression attenuates hyperglycemia-induced accumulation of MG-modified mitochondrial proteins and synthesis of ROS and thus enhances the lifespan of Caenorhabditis elegans [10]. In contrast, inhibition of GlxI reduces the mean and maximum lifespan of the worms. Similarly, overexpression of GlxI reduces hyperglycemia-induced levels of AGEs and oxidative stress in diabetic rats [11]. Importantly, the elevated level of MG in a *Drosophila* model may recapitulate the progression of type 2 diabetes, causing flies to become obese, insulin resistant, and hyperglycemic. This raises the question of whether elevated MG might be a cause of type 2 diabetes [12,13].

The accumulation of MG while cells live in a high concentration of glucose is possibly relevant to diabetes and diabetes complications [14,15,16]. Increases in MG-derived AGEs were also found in experimental and clinical diabetes [17,18]. Furthermore, MG metabolism by the glyoxalase system has been linked to clinical microvascular complications, such as nephropathy, retinopathy, and neuropathy [13,19,20]. Clinical treatment against MG accumulation, such as insulin lispro, has an effect against diabetic nephropathy but only results in a 17% decrease in MG-derived AGEs [21,22,23]. Alternatively, increasing renal GlxI expression also prevented diabetic nephropathy, which is developed along with increased MG glycation [24]. The daily production of MG is about 3 mmol for an adult human, and only about 0.3% of MG forms glycation adducts and the residual MG is removed by GlxI [25]. The amount of MG-derived AGEs variably depends on the rate of expression or degradation of GlxI [16,26,27].

Maintaining a lower MG level is critical for slowing down multiple complications in diabetes [20]. Metformin, prescription medication for treating diabetes, has been reported to be effective in reducing levels of MG and AGEs [28,29]. MG was found to be significantly elevated in diabetic subjects versus the normal control subjects (189.3 ± 38.7 vs. 123.0 ± 37 nmol/L) and was reduced to 158.4 ± 44.2 nmol/L in plasma by taking 1500–2500 mg/day metformin [30]. The scavenging of MG by metformin is related to the reactivity of biguanide toward the dicarbonyl group of MG [20]. The metformin and MG-derived imidazolinone (IMZ), (E)-1,1-dimethyl-2-(5-methyl-4-oxo-4,5-dihydro-1H-imidazol-2-yl)guanidine, was detected in the range of 18.8 nM to 4.3 μM in urine, which may be of therapeutic significance [31]. Alternatively, metformin was found to reduce plasma MG in type 2 diabetic patients by increasing GlxI activity after 24 weeks of medication [28]. Therefore, we speculate that multiple properties of metformin may be responsible for scavenging MG.

In this article, we simultaneously examined the effect of insulin and metformin on cellular MG metabolism and found that a significant increase in cellular MG, S-d-lactoylglutathione, and d-lactic acid was observed in insulin-treated cells. Moreover, MG tended to accumulate in insulin-treated cells by increasing glycolytic leakage and an influx of extracellular MG. The increased MG would result in the accumulation of S-d-lactoylglutathione, which was down-regulated by treating cells with metformin. Since the activity of GlxI and GlxII remained similar in either insulin- or metformin-treated cells, we suggest that MG metabolism may be regulated not just by the activity of GlxI or GlxII under these conditions.

## 2. Materials and Methods

### 2.1. Cell Culture

A human embryonic kidney 293T (HEK293T) cell line was purchased from American Type Culture Collection (ATCC, Manassas, USA). Cells were cultured in Dulbecco’s modified Eagle’s medium (DMEM), a high glucose medium containing 10% fetal bovine serum (FBS) within a 5% CO_2_ atmosphere at 37 °C. Cells of 80% confluence were treated with insulin (Calbiochem, San Diego, CA, United States), metformin, MG, and ^13^C^3^-MG (Sigma-Aldrich, St. Louis, Missouri, United States). 

### 2.2. Cell Lysate Preparation

To each well of a 6-well plate, 100 μL of ultrapure water was added, and 1 × 10^6^ cells were collected into a microtube and disrupted by sonication. After centrifugation at 16,000× *g* for 10 min, the supernatant was subjected to enzyme activity assay, immunoblotting, or LC-ESI-MS analysis.

### 2.3. GlxI Activity Assay

The procedure was performed by following our previous procedures [9]. Monitoring the absorbance of 240 nm by spectrophotometry was applied to measure S-d-lactoylglutathione formation in a GlxI reaction solution containing 8 mM MG, 1 mM glutathione (GSH, Sigma-Aldrich, St. Louis, Missouri, United States), 15 mM MgSO_4_, and 0.2 M imidazole-HCl, pH 7.0 at 25 °C. For 200 μL of reaction, 20 μL of 0.1 μg/μL cell lysate were added to 180 μL of GlxI reaction buffer. GlxI activity was obtained by calculating the slope of absorbance over time of the catalytic velocity curve.

### 2.4. GlxII Activity Assay

The method was modified from a previously described method [32]. The yellow product 5′-thio-2-nitrobenzoic acid (TNB) from reducing 5,5′-dithio-bis(2-nitrobenzoic acid) (DTNB) by GSH was measurable at OD 412 nm. Monitoring the absorbance of 412 nm by spectrophotometry was applied to measure the formation of GSH from S-d-lactoylglutathione hydrolysis in a GlxII reaction solution containing 0.5 mM S-d-lactoylglutathione (Sigma-Aldrich, St. Louis, Missouri, United States) and 0.2 mM DTNB (Sigma-Aldrich, St. Louis, Missouri, United States) in PBS at 25 °C. For 50 μL of reaction, 10 μL of 1 μg/μL cell lysate were added to 40 μL of GlxII reaction buffer. GlxII activity was obtained by calculating the slope of absorbance over time of the catalytic velocity curve.

### 2.5. Immunoblotting

The PVDF membrane was blocked with 3% skim milk in PBS for 1 h and then the protein was recognized by the specific antibody, anti-GlxI (D-5, Santa Cruz biotechnology, Dallas, Texas, USA), anti-GlxII (A-11, Santa Cruz biotechnology, Dallas, Texas, USA), or anti-MG (Cell Biolabs) antibody, in 3 mg/mL of BSA overnight at 4 °C. After washed with PBS three times, each for 10 min, the membrane was further incubated in horseradish peroxidase-conjugated second antibody (PerkinElmer, Waltham, Massachusetts, USA) for 1 h. The signals were detected with the standard ECL protocol (PerkinElmer, Waltham, Massachusetts, USA) after washing the membrane with PBS three times, each for 10 min.

### 2.6. Sample Preparation for LC-ESI-MS Analysis

One volume of collected culture medium or cell lysate was mixed with four volumes of 100% methanol and kept at −80 °C for 30 min. After constant vortexing for 1 min and centrifugation at 16,000× *g* for 10 min at 25 °C, 100 μL of each supernatant were taken for vacuum drying. The dried samples were kept at −20 °C before being subjecting to pre-column derivatization and LC-ESI-MS analysis. S-d-lactoylglutathione was detected in the reconstituted dry sample with 50 μL of ultrapure water by LC-ESI(+)-MS analysis.

### 2.7. Derivatization of MG

The procedures were taken from a previous description except that we used 2,3-diaminopyridine (Sigma-Aldrich, St. Louis, Missouri, United States) instead of o-phenylenediamine in the derivatization reaction [33] and referred to our previous method [9]. The dried samples were reconstituted in 10 μL of 20 mM 2,3-diaminopyridine (Sigma-Aldrich, St. Louis, Missouri, United States) in 2.6 mM formic acid and incubated at room temperature for 24 h. The samples were filled up to 100 μL with ultrapure water and centrifuged at 16,000× *g* for 10 min. The derived samples were subjected to LC-ESI-MS analysis in positive ion mode. The level of MG was determined by detecting the derivative, MG-DAP.

### 2.8. Derivatization of Lactic Acid

The procedures were according to a previous description [34]. For coupling aniline to organic acids, the dried samples were dissolved in 35 μL of ultrapure water and added by 5 μL of 0.3 M aniline (Sigma-Aldrich, St. Louis, Missouri, United States)/HCl (molar ratio: 5/1) and then 5 μL of 20 mg/mL 1-Ethyl-3-(3-dimethylaminopropyl)-carbodiimide (EDC, Sigma-Aldrich, St. Louis, Missouri, United States). The reaction was carried out at room temperature for 2 h and then stopped by adding 5 μL of 10% ammonium hydroxide for a further 30 min incubation. The aniline-derived sample was centrifuged at 16,000× *g* for 10 min. The supernatant was subjected to LC-ESI-MS analysis in negative ion mode. The level of lactic acid was determined by detecting the derivative, lactate-ANL.

### 2.9. LC-ESI-MS Analysis

The LC-ESI-MS system consisted of an ultra-performance liquid chromatography (UPLC) system (ACQUITY UPLC I-Class, Waters, Milford Massachusetts, USA) and an ESI/APCI source of a 4 kDa quadrupole time-of-flight (TOF) mass spectrometer (Waters VION, Waters). The flow rate was set to 0.2 mL/min with the column temperature at 35 °C. Separation was performed with reversed-phase liquid chromatography (RPLC) on a BEH C18 column (2.1 × 100 mm, Waters, Milford Massachusetts, USA) with 7.5 μL sample injection. The elution started from 99% mobile phase A (ultrapure water + 0.1% formic acid) and 99% mobile phase B (100% methanol + 0.1% formic acid), held at 1% B for 0.5 min, raised to 90% B in 5.5 min, held at 90% B for 1 min, and then lowered to 1% B in 1 min. The column was equilibrated by pumping 1% B for 4 min. LC-ESI-MS chromatograms were acquired by positive or negative ion mode under the following conditions: capillary voltage of 2.5 kV, source temperature of 100 °C, desolvation temperature at 250 °C, cone gas maintained at 10 L/h, desolvation gas maintained at 600 L/h, and acquisition by MS^E^ mode with a range of m/z 100-1000 and 0.5 s scan time. The acquired data were processed by UNIFI software (Waters, Milford Massachusetts, USA) with illustrated chromatograms and summarized in an integrated area of signals.

### 2.10. MTS Assay 

Within a 96-well plate, 3 × 10^3^ cells in 100 μL of medium were primed in each well overnight and then treated with MG under various conditions. One volume of MTS (Abcam, Cambridge, UK) was diluted in five volumes of culture medium and 60 μL of mixture were added to each well. After 3–4 h, the developed formazan was measured by a plate reader with an absorbance of 490 nm. The amount of viable cells was evaluated by absorbance readout.

### 2.11. Statistics

Statistical comparisons were performed using a Student’s unpaired, two-tailed *t*-test with results expressed as the mean +/- standard deviation (S.D.). A *p* value of <0.05 was considered statistically significant.

## 3. Results

### 3.1. MG Metabolism Is Affected by Insulin and Metformin

**Figure 1:**
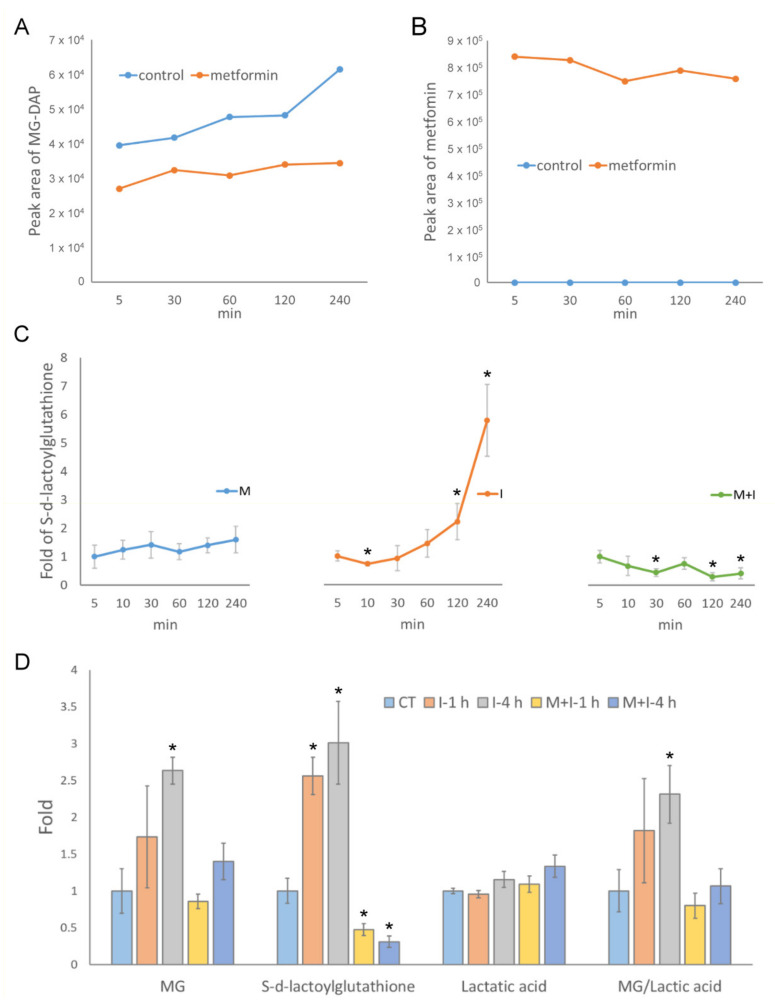
Methylglyoxal (MG) catabolism in metformin- and insulin-treated HEK293T cells. Detection of (**A**) MG derivative (MG-DAP) and (**B**) metformin in culture medium. (**C**) Cellular levels of S-d-lactoylglutathione were detected by LC-ESI-MS in M: 10 mM metformin, I: 10 ng/mL insulin, and M+I: 10 mM metformin + 10 ng/mL insulin-treated HEK293T cells. (**D**) Levels of cellular MG (MG: MG-DAP), S-d-lactoylglutathione, and lactic acid (lactate-ANL) were determined by LC-ESI-MS in HEK293T cells treated with I and M+I for indicated time points after treated conditions. Results significantly different from control at *p* < 0.05 are indicated by * (*n* = 3).

To study the effects of metformin on MG metabolism, we measured the content of MG in culture medium in the presence of 10 mM metformin for 4 h. The content of MG in the medium of metformin-treated HEK293T cells was 55–77% compared to that of untreated cells (Figure 1A), while metformin in the medium remained at much the same level (Figure 1B). With the progression of time, a higher production rate of MG was observed in the medium of untreated cells. Further, we monitored the formation of S-d-lactoylglutathione in metformin-treated HEK293T cells. The results showed that cellular S-d-lactoylglutathione slightly increased with the progression of time under metformin treatment (Figure 1C). Cellular S-d-lactoylglutathione levels significantly increased and reached 2-fold at 2 h and 4.5-fold at 4 h after the administration of insulin. Surprisingly, in metformin/insulin co-treated cells, the levels of S-d-lactoylglutathione dropped to a level less than 30% after 2 h as compared to the initial stage, and the production curve decreased, opposite to those in metformin- or insulin-treated cells (Figure 1C). We then determined the levels of MG, S-d-lactoylglutathione, and lactic acid in insulin- and metformin-treated cells. The results showed that insulin stimulated the accumulation of MG and S-d-lactoylglutathione and retained a higher ratio of MG to lactic acid as compared to untreated cells. In contrast, co-treatment with metformin significantly decreased the levels of MG and S-d-lactoylglutathione as compared to those in insulin-treated cells (Figure 1D). The data indicate that metformin treatment attenuates the efflux of MG, and insulin treatment facilitates the catabolism of MG to S-d-lactoylglutathione. In addition, co-treatment with metformin in insulin-treated cells greatly increases the conversion of MG to S-d-lactoylglutathione and possibly to lactic acid.

### 3.2. Modulation of GlxI and GlxII Activity by Insulin and Metformin

**Figure 2:**
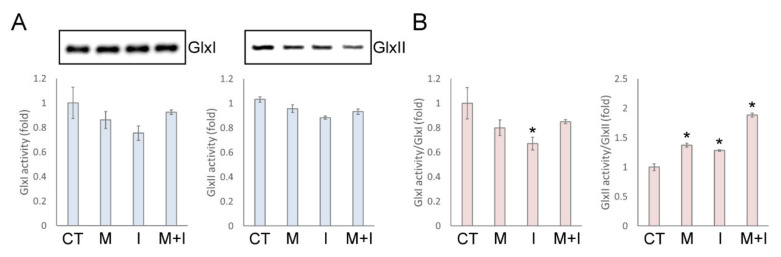
The individual activity and protein levels of glyoxalase system under insulin and metformin treatment. (**A**) The activity and enzyme expression level, and (**B**) normalized activity of glyoxalase 1 (GlxI) and GlxII of whole cell lysates from insulin- and metformin-treated HEK293T cells. Results significantly different from control at *p* < 0.05 are indicated by * (*n* = 3).

We then examined the activity and protein level of GlxI and GlxII under insulin and metformin treatment. As shown in Figure 2A, GlxI and GlxII activity remained much the same level in whole cell lysates under insulin or metformin treatment. However, insulin induced up-regulation of GlxI and down-regulation of the GlxII protein level in the presence or absence of metformin (Figure 2A). Then, the activity was normalized as the ratio of the whole enzyme activity to the intensity of the immunoblot signals. The normalized GlxI activity was decreased, and the normalized GlxII activity was increased with metformin, insulin, and insulin/metformin co-treatment (Figure 2B). However, the change of GlxI and GlxII activity in cells treated with metformin and insulin is relatively small as compared with the changes in MG and S-d-lactoylglutathione levels (Figure 1), and other factors may contribute to the changes in MG metabolism under insulin and metformin actions.

### 3.3. Protection of Cells from MG Assaults by Insulin and Metformin

**Figure 3:**
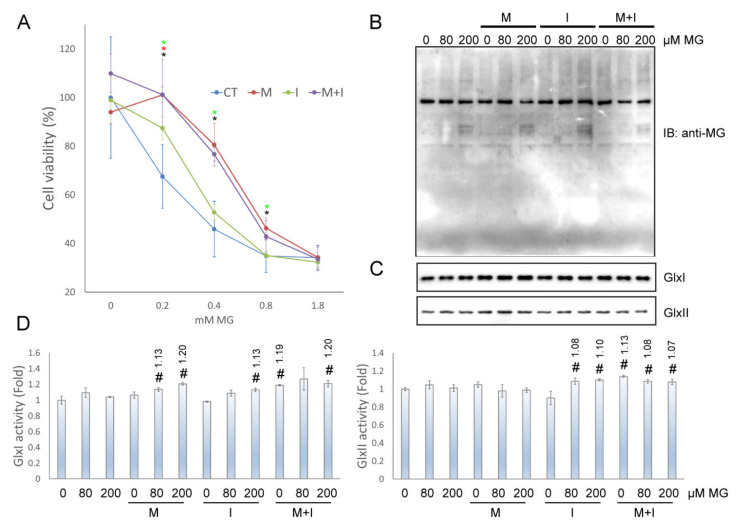
The effects of insulin and metformin on MG assaults in HEK293T cells. (**A**) Viability of HEK293T cells cultured at various concentrations of MG in the absence or presence of insulin and metformin for 4 h. (**B**) AGE formation, (**C**) enzyme level of GlxI and GlxII, and (**D**) enzyme activity of GlxI and GlxII in HEK293T cells at various MG concentrations in the absence or presence of insulin and metformin for 4 h. MG: MG. M: 10 mM metformin; I: 10 ng/mL insulin; M+I: 10 mM metformin + 10 ng/mL insulin. Results with significant difference at *p* < 0.05 are indicated by * metformin treated cells, * insulin treated cells, and * metformin/insulin co-treated cells compared to control cells under the same MG concentration (*n* = 4). Enzyme activity significantly different from control at *p* < 0.05 is indicated by # (*n* = 3).

Although insulin and metformin were involved in the regulation of intracellular MG metabolism, the actions on MG transmembrane transport remains unknown. The MTS assay was performed to study the effects of insulin and metformin against the assaults induced by extracellularly applied MG. The results showed that insulin, metformin, or metformin/insulin co-treatment would prevent the cells from dying at toxic concentrations of 0.2, 0.4, and 0.8 mM MG for a 4 h incubation (Figure 3A). However, metformin treatment and metformin/insulin co-treatment were much potent than the insulin treatment. In addition, we observed the formation of MG-induced AGEs at various concentrations of MG in the presence of insulin and metformin. The formation of AGEs was suppressed by metformin whether insulin was added or not. Interestingly, insulin actually enhanced the accumulation of AGEs (Figure 3B). Under these conditions, the expression and activity of GlxI and GlxII changed to a limited extent, 20% for GlxI and 13% for GlxII at most (Figure 3C and 3D). The results imply that other mechanisms than GlxI and GlxII may be responsible for the disposal of extracellular MG. 

### 3.4. Use of ^13^C_3_-MG for the Study of MG Metabolism

**Figure 4:**
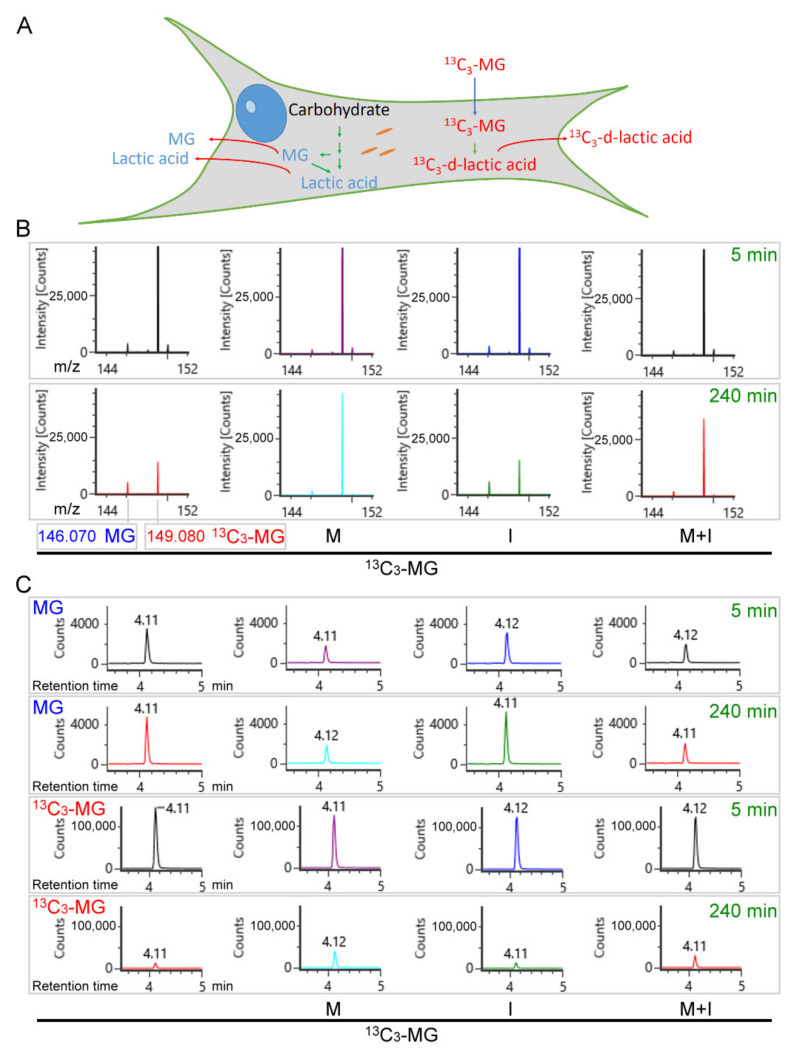
Using isotopic ^13^C_3_-MG to track the metabolism of extracellular MG. (**A**) The related intermediates from MG or carbohydrate are derived from either intracellular or extracellular origin. (**B**) Mass spectrum and (**C**) chromatogram of methylglyoxal (MG) and ^13^C_3_-MG were acquired by LC-ESI-MS analysis of the medium collected from HEK293T cell culture with the indicated conditions. M: 10 mM metformin; I: 10 ng/mL insulin; M + I: 10 mM metformin + 10 ng/mL insulin; ^13^C_3_-MG: 80 μM ^13^C_3_-MG.

To further understand the fate of extracellular MG, we introduced the stable isotopic MG (^13^C_3_-MG) for tracking the downstream intermediates of MG. Intracellular ^13^C_3_-MG and in situ production of ^13^C_3_-lactic acid can be measured. Meanwhile, the consumption of ^13^C_3_-MG in culture medium can also be monitored (Figure 4A). After administration of ^13^C_3_-MG, culture media and cells were collected, respectively, for the analysis of MG, ^13^C_3_-MG, lactic acid, and ^13^C_3_-lactic acid under insulin or metformin treatment. Metabolites were detected by acquiring the mass signal of m/z 146.070 ± 5 ppm for the derivative of MG ([MG-DAP+H]^+^), m/z 149.080 ± 5 ppm for ^13^C_3_-MG ([^13^C_3_-MG-DAP+H]^+^), m/z 164.070 ± 5 ppm for lactic acid ([lactate-ANL+H]^+^), and m/z 167.080 ± 5 ppm for ^13^C_3_-lactic acid ([^13^C_3_-lactate-ANL+H]^+^). Then, all detectable isotopic intermediates relative to MG were calculated for estimating the efficiency of the glyoxalase system in culture medium and cell lysates.

### 3.5. Inhibition of MG Transport across Plasma Membrane by Metformin 

**Figure 5:**
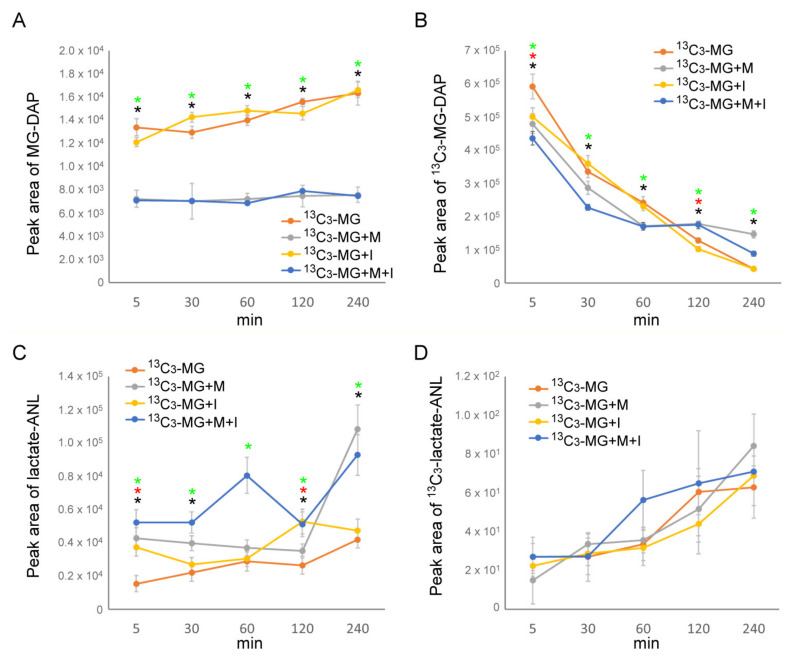
The change of MG and lactic acid in media from insulin- and metformin-treated HEK293T cells. The levels of MG (**A**) MG-DAP, (**B**) ^13^C_3_-MG-DAP, (**C**) lactic acid, and (**D**) ^13^C_3_-lactate-ANL in media were determined by LC-ESI-MS by applying ^13^C_3_-MG for tracking within 4 h incubation. ^13^C_3_-MG: 80 μM ^13^C_3_-MG; ^13^C_3_-MG+M: 80 μM ^13^C_3_-MG + 10 mM metformin; ^13^C_3_-MG+I: 80 μM ^13^C_3_-MG + 10 ng/mL insulin; ^13^C_3_-MG+M+I: 80 μM ^13^C_3_-MG + 10 mM metformin + 10 ng/mL insulin. Results with a significant difference at *p* < 0.05 are indicated by * ^13^C_3_-MG+M, * ^13^C_3_-MG+I, and * ^13^C_3_-MG+M+I compared to ^13^C_3_-MG-treated cells (*n* = 3).

According to the acquired mass spectrum, whether in the presence of insulin or not, metformin maintained the mass peak of *m*/*z* 146.070 ± 5 ppm ([MG-DAP+H]^+^) at a lower level at all times as compared to untreated and insulin-treated cell media. Moreover, metformin treatment or insulin/metformin co-treatment maintained the mass peak of *m*/*z* 149.080 ± 5 ppm ([^13^C_3_-MG-DAP+H]^+^) at a higher level after 4 h incubation compared to untreated and insulin-treated cell media (Figure 4B). From the semi-quantitative results, the secreted MG ([MG-DAP+H]^+^) was monitored in culture medium by integrating the LC-ESI-MS peak of the extracted ion chromatogram (EIC m/z: 146.070 ± 5 ppm) (Figure 4C) and is summarized in Figure 5A. The consumption of ^13^C_3_-MG was monitored in culture medium by integrating the LC-ESI-MS peak of EIC m/z: 149.080 ± 5 ppm (Figure 4C) and is summarized in Figure 5B. In the presence of insulin or not, metformin maintained the secreted MG at about 50% of the level of untreated or insulin-treated cell media at all times of treatment (Figure 5A). Meanwhile, consumption of extracellular ^13^C_3_-MG was slowed down after 2 h of incubation with metformin despite the presence of insulin (Figure 5B). The results suggest that metformin could suppress the transmembrane transport of MG, either influx (^13^C_3_-MG) or efflux (MG). On the other hand, insulin exerts little effect on MG transport.

### 3.6. The Production and Secretion of Lactic Acid Facilitated by Insulin and Metformin

To evaluate the turnover of MG, we monitored the levels of extracellular lactic acid with LC-ESI-MS. By the same process described in Section 3.5 and Figure 4, the results were obtained by applying signals m/z 164.070 ± 5 ppm for lactic acid ([lactate-ANL+H]^+^) and m/z 167.080 ± 5 ppm for ^13^C_3_-lactic acid ([^13^C_3_-lactate-ANL+H]^+^). We found that both insulin and metformin could facilitate the production and secretion of lactic acid, which comes from intracellular carbon sources (Figure 5C) while only a small increase in ^13^C_3_-lactate-ANL was observed in the metformin-containing medium (Figure 5D). By calculating the slope of ^13^C_3_-lactate-ANL vs. time for 4 h, the rates of ^13^C_3_-lactate-ANL formation were obtained as 1.56-fold, 1.13-fold, and 1.10-fold for metformin, insulin, and metformin/insulin co-treatment, respectively, as compared to the untreated cells. 

### 3.7. Insulin Directs MG toward the Glyoxalase System

**Figure 6:**
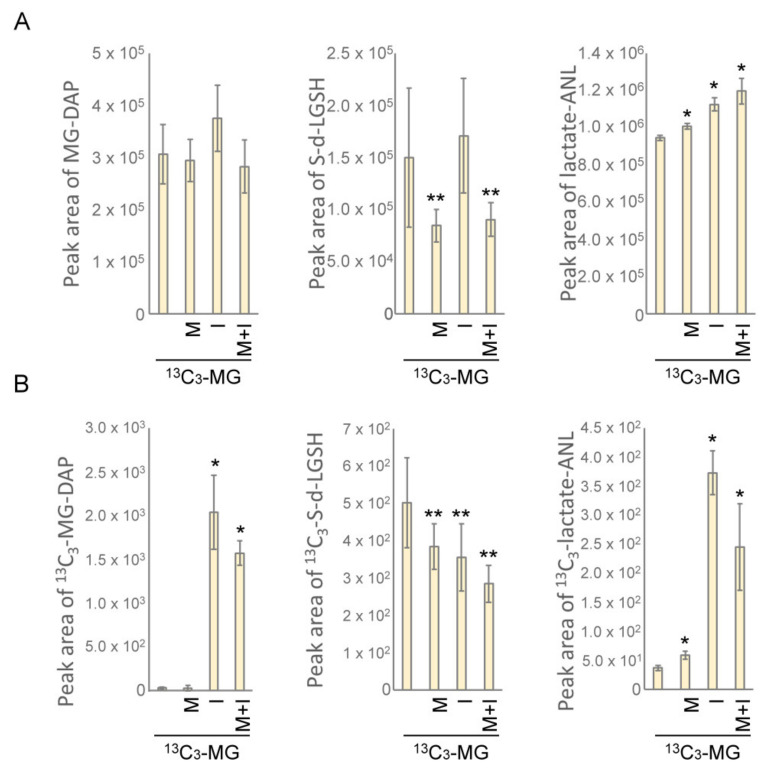
The change of intracellular MG, S-d-lactoylglutathione, and lactic acid in insulin- and metformin-treated HEK293T cells. The levels of (**A**) MG (MG-DAP), S-d-lactoylglutathione, and lactic acid (lactate-ANL) from glucose catabolism and (**B**) ^13^C_3_-MG-DAP, ^13^C_3_-S-d-lactoylglutathione, and ^13^C_3_-lactate-ANL from added ^13^C_3_-MG were determined by LC-ESI-MS by applying 80 μM ^13^C_3_-MG (^13^C_3_-MG) in medium for tracking with 4 h incubation. M: 10 mM metformin; I: 10 ng/mL insulin; M+I: 10 mM metformin + 10 ng/mL insulin. Results significantly different from control at *p* < 0.05 are indicated by * (*n* = 3) and ** (*n* = 9).

The accumulation of S-d-lactoylglutathione and MG resulting from vigorous glucose metabolism under insulin treatment (Figure 1C,D) would cause overloading of the glyoxalase system. However, insulin actually protected cells from MG assault (Figure 3A). With ^13^C_3_-MG tracking, we surprisingly found that about a 60-fold increase in intracellular ^13^C_3_-MG was measured in the presence of insulin as compared to the untreated or metformin-treated cells. Meanwhile, the changes in the endogenous MG level remained similar to those cells without extracellular ^13^C_3_-MG application (Figure 6A and Figure 1D). For further tracking of the influx of ^13^C_3_-MG, we also acquired the profiles of isotopic S-d-lactoylglutathione. The levels of ^13^C_3_-S-d-lactoylglutathione in metformin and insulin-treated cells decreased as compared to that in untreated cells (Figure 6B). However, the levels of S-d-lactoylglutathione in insulin-treated cells increased as compared to that in untreated cells (see also Figure 1D). Then, we surveyed the product of the glyoxalase system, and a robust increase in ^13^C_3_-lactate-ANL was observed, which is similar to the pattern of internalized ^13^C_3_-MG (Figure 6). Metformin treatment also facilitated the production of intracellular ^13^C_3_-lactic acid (Figure 6B) although ^13^C_3_-MG uptake is limited by cells (Figure 5A). The results indicated that insulin may direct extracellular MG to the glyoxalase system.

### 3.8. Metformin Maintains Cellular Redox State by Generating Reduced Glutathione

**Figure 7:**
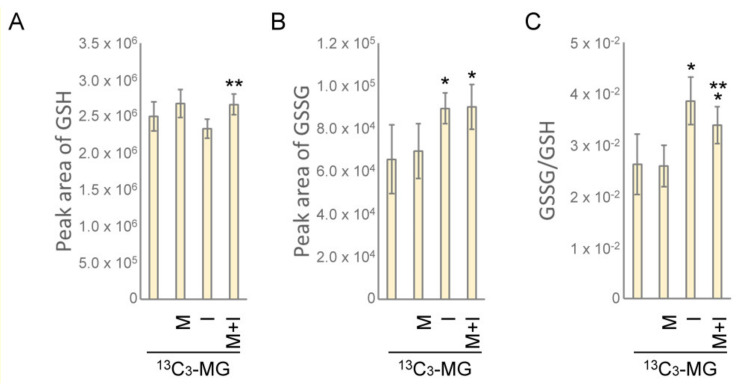
The change of intracellular reduced and oxidized glutathione in insulin- and metformin-treated HEK293T cells. The intracellular levels of (**A**) reduced glutathione (GSH) and (**B**) oxidized glutathione (GSSG) and (**C**) the ratio of GSSG/GSH were determined by LC-ESI-MS. Cells were treated with M: 10 mM metformin; I: 10 ng/mL insulin; M+I: 10 mM metformin + 10 ng/mL insulin in the presence of 80 μM ^13^C_3_-MG (^13^C_3_-MG) for 4 h. Results significantly different from control and I at *p* < 0.05 are, respectively, indicated by * and ** (*n* = 9).

Finally, we examined the change of glutathione species in HEK293T cells treated with insulin and metformin in the presence of ^13^C_3_-MG. The level of reduced glutathione slightly increased in metformin-treated cells but decreased in insulin-treated cells. However, the insulin-induced decrease in glutathione level was significantly recovered in insulin/metformin co-treated cells (Figure 7A). Moreover, oxidized glutathione was significantly increased in insulin-treated cells or metformin/insulin co-treated cells (Figure 7B). The overall redox state of glutathione was then expressed by the ratio of GSSG/GSH. As shown in Figure 7C, insulin stimulation resulted in high ratios of GSSG/GSH, however, metformin/insulin co-treatment significantly lowered the ratio as compared to insulin treatment. Therefore, the results indicated that insulin treatment facilitates the catabolism of MG to S-d-lactoylglutathione at the cost of reduced glutathione. On the other hand, the metformin-induced formation of reduced glutathione maintains the cellular redox state and supports the sustained formation of MG-glutathione hemithioacetal.

## 4. Discussion

Metformin is known to scavenge MG in circulation by forming adducts with MG, such as IMZ [31] and also up-regulate GlxI activity after medication for 24 weeks [28]. In this study, we have resolved the cellular MG metabolism by tracking the origin of the related intermediates and examining the function of the glyoxalase system. From the results, metformin actually suppressed the efflux of intracellular MG (Figure 1A and Figure 5A) and the influx of extracellular ^13^C_3_-MG from media (Figure 4C and Figure 5B). Moreover, metformin facilitated the catabolism of MG and S-d-lactoylglutathione (Figure 1C,D and Figure 6A,B). The combined effects of metformin will maintain a lower concentration of intracellular MG. However, GlxI activity was not significantly up-regulated under these conditions (Figure 2). Therefore, other unknown mechanisms regulating the glyoxalase system by metformin may be involved.

Cell metabolism in a high glucose concentration results in the accumulation of MG and is relevant to diabetes and the associated complications [14,15,16]. Most MG comes from glycolytic leakage and is regulated by insulin signaling. We found that MG and S-d-lactoylglutathione in HEK293T cells were robustly formed during insulin stimulation (Figure 1D and Figure 6A). Surprisingly, an accelerated influx of extracellular ^13^C_3_-MG was provoked and synchronously converted into ^13^C_3_-lactic acid (Figure 6B) under insulin treatment. The results indicate that insulin stimulation tends to increase intracellular MG by enhancing glucose metabolism and also influx of the extracellular MG. Meanwhile, an increase in MG modification of cellular proteins was also detected in insulin-treated HEK293T cells (Figure 3B). Since MG is known to impair insulin signaling and induce insulin resistance [35,36], prolonged insulin stimulation, such as diabetes-induced hyperinsulinemia, would result in the overproduction of MG.

Since the level of MG is linked to clinical microvascular complications, such as nephropathy, retinopathy, and neuropathy [13,19,20], the administration of drugs facilitating MG removal are theoretically able to reduce the associated pathogenesis. Both metformin and insulin treatment protected cells from MG assaults (Figure 3A). In the view of insulin-induced metabolism change, a bailout is needed in response to higher levels of MG shunted from elevated glycolytic flux. From the metabolic profiling, we confirmed that both insulin and metformin promote the production of cellular lactic acid from carbohydrate catabolism (Figure 5C and Figure 6B). In addition, a higher ratio of intracellular ^13^C_3_-lactic acid was generated in insulin-treated cells (Figure 6B). All these results imply that the levels of MG and the glyoxalase system are also regulated by insulin.

By monitoring ^13^C_3_-MG catabolism, a very low content of intracellular and extracellular ^13^C_3_-S-d-lactoylglutathione or ^13^C_3_-lactic acid was detected in all the conditions with 4 h of culture after adding ^13^C_3_-MG (Figure 5D and Figure 6B). Meanwhile, significant increases in intracellular lactic acid were observed in insulin- and metformin-treated cells (Figure 6A), and metformin appeared to facilitate the efflux of intracellular lactic acid at certain time points of treatment (Figure 1D and Figure 5C). From these results, we suggest that metformin maintains the intracellular MG by improving the efficiency of the glyoxalase system and limiting both the influx and efflux of MG. The efficiency of the glyoxalase system can be improved by coordinated interaction of GlxI and GlxII or by elevation of intracellular reduced glutathione levels (Figure 7). The redox homeostasis and the supply of reduced glutathione in the presence of metformin contribute to the improved efficiency of the glyoxalase system. How metformin hinders the membrane transport of MG remains elusive. Since both the influx and efflux of MG are suppressed by metformin treatment, it is highly possible that metformin exerts a direct effect on the MG transporter. A parasitic protozoan aquaglyceroporin, LmAQP1, is permeable to water, glycerol, MG, and dihydroxyacetone [37]. It is worthwhile to study whether mammalian aquaglyceroporins are permeable to MG and sensitive to metformin.

Interestingly, metformin was found to increase GlxI activity in peripheral blood mononuclear cells and red blood cells in type 2 diabetic patients after 24 weeks of medication [28]. On the other hand, metformin was also found to suppress the expression and activity of GlxI in many types of cancer cells [38,39,40]. These studies indicate that metformin tends to inhibit GlxI expression/activity in cancer cells and increase GlxI expression/activity in noncancerous cells with long-term treatment. In our findings, GlxI expression/activity remained at much the same levels while HEK293T cells were treated with metformin in various conditions for 4 h. The time of treatment with metformin may be insufficient to affect GlxI expression. However, metformin provokes a transient effect on facilitating MG metabolism during insulin stimulation, which is beneficial to cells to prevent dicarbonyl stress.

## 5. Conclusions

**Figure 8:**
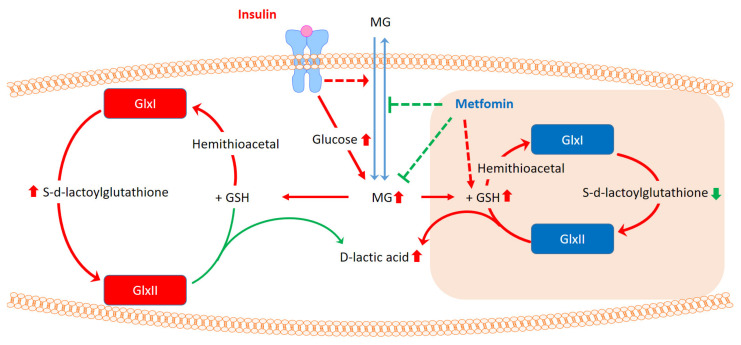
The coordinated effects of insulin and metformin on the glyoxalase system for maintaining intracellular concentrations of MG.

In this article, we disclose an alternative biochemical regulation of the glyoxalase system as the proposed model in Figure 8. Under vigorous carbohydrate metabolism, MG and related intermediates are accumulated due to the insufficient capacity of the glyoxalase system. However, cells simultaneously allow the influx of extracellular MG in response to insulin signaling. Insulin treatment facilitates the catabolism of MG to S-d-lactoylglutathione at the cost of reduced glutathione. With an unknown mechanism coordinating GlxI and GlxII to improve the efficiency of the glyoxalase system, metformin facilitates MG catabolism and the pumping out of lactic acid. In addition, metformin also limits the transport of MG in and out of cells and maintains the redox state of glutathione under dicarbonyl stress. The lowered intracellular MG is beneficial to insulin treatment. 

## Data Availability

Data is contained within the article.
